# Evidence for Conserved Function of γ–Glutamyltranspeptidase in *Helicobacter* Genus

**DOI:** 10.1371/journal.pone.0030543

**Published:** 2012-02-14

**Authors:** Mirko Rossi, Christian Bolz, Joana Revez, Sundus Javed, Nahed El-Najjar, Florian Anderl, Heidi Hyytiäinen, Pia Vuorela, Markus Gerhard, Marja-Liisa Hänninen

**Affiliations:** 1 Department of Food Hygiene and Environmental Health, Faculty of Veterinary Medicine, University of Helsinki, Helsinki, Finland; 2 Department of Medical Microbiology, Immunology and Hygiene, Technische Universität München, Munich, Germany; 3 Pharmaceutical Sciences, Department of Biosciences, Abo Akademi University, Turku, Finland; University of Edinburgh, United Kingdom

## Abstract

The confounding consequences of *Helicobacter bilis* infection in experimental mice populations are well recognized, but the role of this bacterium in human diseases is less known. Limited data are available on virulence determinants of this species. In *Helicobacter pylori*, γ-glutamyltranspeptidase (γGT) contributes to the colonization of the gastric mucosa and to the pathogenesis of peptic ulcer. The role of γGT in *H. bilis* infections remains unknown. The annotated genome sequence of *H. bilis* revealed two putative *ggt* genes and our aim was to characterize these *H. bilis* γGT paralogues. We performed a phylogenetic analysis to understand the evolution of *Helicobacter* γGTs and to predict functional activities of these two genes. In addition, both copies of *H. bilis* γGTs were expressed as recombinant proteins and their biochemical characteristics were analysed. Functional complementation of *Esherichia coli* deficient in γGT activity and deletion of γGT in *H. bilis* were performed. Finally, the inhibitory effect of T-cell and gastric cell proliferation by *H. bilis* γGT was assessed. Our results indicated that one gene is responsible for γGT activity, while the other showed no γGT activity due to lack of autoprocessing. Although both *H. bilis* and *H. pylori* γGTs exhibited a similar affinity to L-Glutamine and γ-Glutamyl-p-nitroanilide, the *H. bilis* γGT was significantly less active. Nevertheless, *H. bilis* γGT inhibited T-cell proliferation at a similar level to that observed for *H. pylori*. Finally, we showed a similar suppressive influence of both *H. bilis* and *H. pylori* γGTs on AGS cell proliferation mediated by an apoptosis-independent mechanism. Our data suggest a conserved function of γGT in the *Helicobacter* genus. Since γGT is present only in a few enterohepatic *Helicobacter* species, its expression appears not to be essential for colonization of the lower gastrointestinal tract, but it could provide metabolic advantages in colonization capability of different niches.

## Introduction

γ-Glutamyltranspeptidase (γGT) is a threonine N-terminal nucleophile (Ntn) hydrolase that catalyses the transpeptidation and hydrolysis of the γ-glutamyl group of glutathione and related compounds [1]. γGT is widely distributed in living organisms and is highly conserved, with mammalian and bacterial homologues often sharing more than 25% of sequence identity [2]. From the ∼1000 of whole genome sequenced bacterial species available in MEROPS databases (http://merops.sanger.ac.uk [3]), 540 (∼200 genera) possess γGT-like proteins belonging to protease family T03. Moreover, several bacterial species carry multiple copies of genes annotated as γGT, but the majority of these genes lack functional verification.

γGT is found in all gastric *Helicobacter* species. However, among the 20 validly published enterohepatic *Helicobacter* species (EHS), only *H. aurati*, *H. bilis*, *H. canis*, *H. muridarum* and *H. trogontum* express this enzyme [4]. In *H. pylori*, γGT represents an important virulence factor, playing a role in colonization [5], [6] and pathogenesis [7]–[10]. It is constitutively expressed *in vivo* and *in vitro* [11] and enables the bacterial cells to use extracellular glutamine and glutathione as a source of glutamate [9], [12]. Purified Hp-γGT was shown to be involved in upregulation of growth factors in MKN-28 gastric cells [7], to induce apoptosis in AGS cells [10], to inhibit T-cell proliferation [9] and to contribute to *H. pylori*–mediated H_2_O_2_ generation [8]. By contrast, the biochemical properties of EHS γGT and its role in the colonization of the gut and in the pathogenesis of gastrointestinal and hepatobiliary diseases are completely unknown. EHS is a phenotypically and genotypically heterogeneous phylogroup within the *Helicobacter* genus [4], including species usually colonizing the intestinal tract and/or the liver of mammals and birds. Although EHS could be considered part of the normal microbiota of rodents, some species cause diseases in these animals [13]. In particular, *H bilis*, an endemic EHS in most experimental mouse colonies, induces disease in susceptible animals and may substantially confound interpretations of some research studies [14]. Natural *H. bilis* infection in inbred [15] or outbred mice [16] has been associated with multifocal hepatitis. Moreover, *H. bilis* has been used experimentally to induce inflammatory bowel disease (IBD) in mdr^−/−^ and IL-10^−/−^ knock-out mice [16], typhlocolitis in the C3H/HeN mice strain [17] and cholesterol gallstone formation in C57L mice [18]. *H. bilis* is able to infect and cause diseases in different animal hosts, showing one of the broadest host spectrums in the *Helicobacter* genus [19]. It was isolated from the aborted fetus of sheep and pig [19] and from chronic hepatobiliary diseases in hamsters [20]. *H. bilis* has been also isolated from human patients with chronic diarrhoea [21] and pyoderma gangrenosum-like ulcers [22]. In addition, several studies have reported an association of this species with chronic liver diseases [23], [24] or biliary tract and gallbladder cancers [25], [26] in human, using either PCR or serological tests. Limited data are available on virulence determinants of *H. bilis* [27]–[29], and no studies to date have described the biochemical and biological proprieties of *H. bilis* γGT (Hb-γGT).

In contrast to observations in gastric *Helicobacter* spp., the genome sequence of *H. bilis* ATCC 43879 revealed the presence of two *ggt* copies. In this study, we used a phylogenetic and a functional approach to analyse both *H. bilis* γGT paralogues. Although both genes were phylogenetically related to other *Helicobacter* γGTs, analysis of the recombinant proteins, western blot using specific antibodies, complementation of *E. coli* Δ*ggt* and mutation in *H. bilis* clearly showed that only one gene was responsible for *H. bilis* γGT activity. The γGT of *H. bilis* exhibited a similar affinity as *H. pylori* to γ-Glutamyl-p-nitroanilide and to L-Glutamine; however, it was significantly less active. Nevertheless, *H. bilis* γGT inhibited T-cell and gastric cell proliferation at a similar level to that observed for *H. pylori* γGT. The inhibition observed was mediated by an apoptosis-independent mechanism and suggested a conserved function of γGT in *Helicobacter* genus.

## Results

### Sequence analysis revealed marked differences between two γGT paralogues of *H. bilis* ATCC 43879

**Figure 1:**
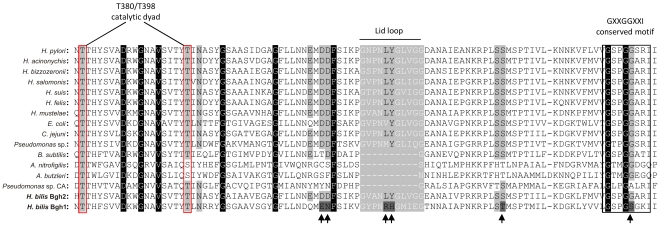
Multi-alignment of amino acid sequences of different bacterial γGTs, *Helicobacter bilis* Bgh1 and Bgh2, and class IV Cephalosporin Acylase (CA). Multi-alignment between residues 379 and 477 (*Helicobacter pylori* γGT numeration) is shown. Sequences of γGTs of *H. pylori* (26695; HP1118), *H. acinonychis* (Sheeba; Hac_0598), *H. bizzozeronii* (CIII-1;HBZC1_08080), *H. salomonis* (O6A; EMBL FR821684), *H. suis* (HS1; HSUHS1_0265), *H. felis* (ATCC 49179; Hfelis_06880), *H. mustelae* (12198; HMU08020), *Esherichia coli* (K12; Swiss-Prot P18956), *Campylobacter jejuni* (81–176; CJJ81176_0067), *Pseudomonas* sp. (A14; Swiss-Prot P36267), *Bacillus subtilis* (168; Swiss-Prot P54422), *Arcobacter nitrofigilis* (DSM 7299; Arnit_0203), *Arcobacter butzleri* (RM4018; Abu_0961), *H. bilis* (Bgh1 = HRAG_01341; Bgh2 = HRAG_01828) as well as class IV CA of *Pseudomonas* sp. (SE82; Swiss-Prot P15557) are shown. Residues completely conserved among the sequences are indicated with a black background. The catalytic dyad is highlighted with a red box, and the conserved motif GXXGGXXI is enclosed in a black box. The Lid loop consists of the residues G428 to G438 of *H. pylori* γGT and is indicated in grey. Residues involved in the substrate recognition and the catalytic centre are highlighted in grey. Amino acid substitutions in Bgh1 potentially involved in functional change are indicated by arrows below the sequences.

The *ggt* paralogues HRAG_01341 and HRAG_01828 of the human-associated *H. bilis* strain ATCC 43879 genome (NCBI ACDN0000000), were named *bgh1* (*H. bilis ggt* homologue 1) and *bgh2* (*H. bilis ggt* homologue 2), respectively. Nucleotide and amino acid similarity between the two *H. bilis* γGT paralogues, and between each homologue and *H. pylori* γGT (Hp-γGT; HP1118) were analysed by pairwise global alignment. The two *H. bilis* γGT paralogues showed 65.8% and 62.0% of nucleotide and amino acid identity, respectively. Moreover, in comparison with Hp-γGT, Bgh2 showed 65.2% of amino acid identity, while Bgh1 showed 53.4%. To detect amino acid positions potentially involved in functional change, conserved sites of both Bgh1 and Bgh2 genes were evaluated on the basis of γGT structural data available for *H. pylori* [30], [31] and *E. coli* [32]. A multi-alignment including all *Helicobacter* γGT sequences, other bacterial γGTs and class IV cephalosporin acylase (CA) of *Pseudomonas* sp. strain SE82, was constructed (Figure 1). In Bgh2, all the functional sites described for *H. pylori* are conserved. In Bgh1, by contrast, amino acid substitutions in the 20 kDa subunit potentially involved in functional change were observed. The substitutions were as follows: Asp421Glu, Asp422Asn, Leu432Arg, Tyr433His, Ser452Thr and Gly473Ser (Hp-γGT numeration).

To predict the localization of both *H. bilis* γGT paralogues, the sequences were submitted to SignalP 3.0, using both Neural networks and Hidden Markov Models (HMM) methods [33], PSLpred (Hybrid Approach Based; [34]), PSORTb v3.0.2 [35] and CELLO v2.5 [36]. These tools were unable to uniformly predict the sub-cellular localization of both Bgh1 and Bgh2. To verify the presence of other potential start codons, we analysed the upstream region of both genes. We identified a second putative start codon 54 bp upstream of the annotated methionine of Bgh2, but no other potential start codons for Bgh1. Using the new predicted start codon, all of the analysis strongly predicted Bgh2 as a periplasmic protein, and SignalP detected the presence of *sec* signal peptide sequence with a potential cleavage site between positions 30 and 31 (VFA-AS). The same prediction was obtained for all γGTs of *Helicobacter* spp. and *C. jejuni*. No twin-arginine signal peptide cleavage site was detected in any γGTs of *Helicobacter* spp. and *C. jejuni*.

### Phylogenetic analysis of *Helicobacter* γGTs

**Figure 2:**
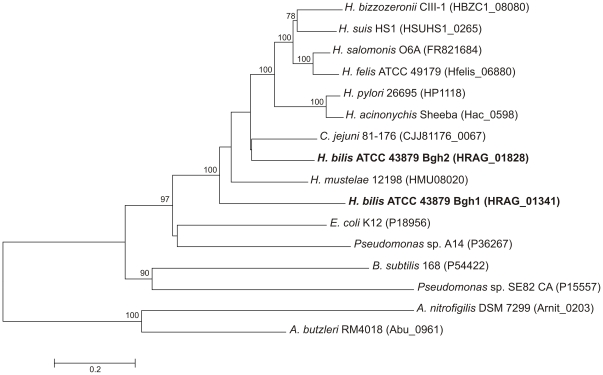
Unrooted tree based on complete amino acid sequences of different bacterial γGTs, *Helicobacter bilis* Bgh1 and Bgh2, and *Pseudomonas* Cephalosporin Acylase (CA). The evolutionary history was inferred using the Minimum Evolution method and the evolutionary distances were computed using the Dayhoff matrix-based method. Bar indicates amino acid substitutions per position. Numbers at the nodes indicate support for the internal branches within the tree obtained by bootstrap analysis (≥70%; percentages of 500 bootstraps).

Phylogenetic relationships among epsilon proteobacteria and other bacterial γGTs and *Pseudomonas* spp. CA were analysed (Figure 2). The Minimum Evolution tree based on amino acid sequence alignment showed that both *H. bilis* γGT paralogues cluster with *Helicobacter* and *C. jejuni* γGTs, forming, however, two distinct branches: Bgh2 and *C. jejuni* γGT belonged to a single clade, while Bgh1 formed a separate branch.

**Figure 3:**
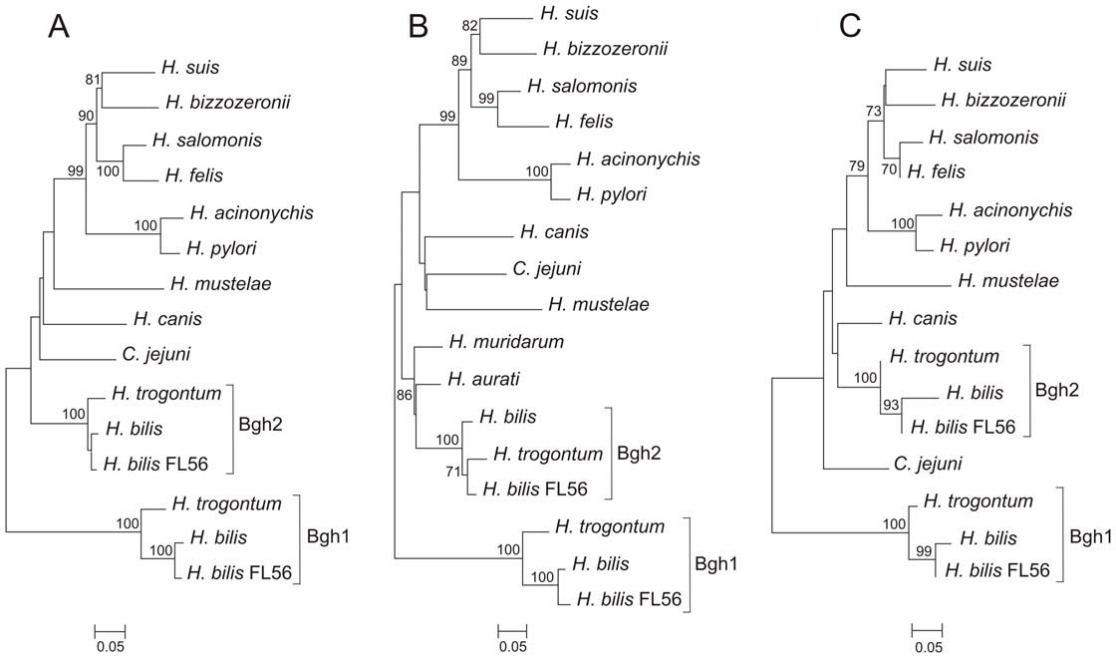
Unrooted tree based on the amino acid sequences of: *Helicobacter* spp. γGTs, *Campylobacter jejuni* γGTs and Bgh1 and Bgh2 homologues of *Helicobacter trogontum* and *Helicobacter bilis*. The evolutionary history was inferred using the Minimum Evolution method, and the evolutionary distances were computed using the Dayhoff matrix-based method. Bars indicate amino acid substitutions per position. Numbers at the nodes indicate support for the internal branches within the tree obtained by bootstrap analysis (≥70%; percentages of 500 bootstraps). (A) Phylogeny of almost complete pro-enzyme sequences (447 AA). (B) Phylogeny of almost complete N-terminal γGT sequences (Heavy chain; 333 AA). (C) Phylogeny of the almost complete C-terminal γGT sequences (Light chain; 190 AA).

To examine the phylogenetic relationships among *Helicobacter* γGTs in more detail and to identify other *Helicobacter* species potentially carrying multiple γGT sequences, consensus degenerate hybrid oligonucleotide primers were designed on the basis of highly conserved amino acid motifs (Table 1). A fragment of about 1300 bp, corresponding to an almost complete *ggt* sequence, was successfully amplified from *H. canis*, and two fragments, corresponding to an almost complete sequence of two distinct *ggt* copies, were amplified from *H. trogontum* and *H. bilis* genomospecies FL56. From *H. aurati* and *H. muridarum*, amplification of only the N-terminal part of a single *ggt* copy was possible. An unrooted Minimum Evolution tree was built on the basis of an almost complete amino acid sequence of the γGT pro-enzyme of *Helicobacter* spp. and *C. jejuni* (Figure 3a). Phylogenetic analysis showed that the two *ggt* copies amplified from *H. trogontum* and *H. bilis* genomospecies FL56 corresponded to orthologues of *bgh1* and *bgh2*, whereas the sequences obtained from *H. canis*, *H. aurati* and *H. muridarum* (Figure 3a,b) clustered with other *Helicobacter ggt* and *bgh2*. In Figure 3, the phylogeny of the pro-enzyme was compared with those of the single sub-units. The trees showed differences in both the topology and the bootstrap supporting nodes, suggesting different evolution of the two parts. The same results were obtained comparing the maximum likelihood trees of the nucleotide sequences (data not shown). Finally, to provide a measure for the selective pressure that the gene pair *bgh1* and *bgh2* was subjected to, the ratio between non-synonymous/synonymous substitution (Ka∶Ks) was evaluated by sliding window analysis (window size 50 bp) using SWAKK [37]. A Ka∶Ks ratio smaller than one was observed in all positions analysed. The average Ka∶Ks ratio was calculated to be 0.175, indicating that the genes are under purifying selection. A similar Ka∶Ks ratio was observed also for the *bgh1* and *bgh2* pairs of *H. trogontum* and *H. bilis* genomospecies FL56 (data not shown).

**Table 1:** Oligonucleotides used in this study.

Oligonucleotides	Sequence
ggtCODEHOPfw-b	GATGAAGGCGGGAATGCTathgaygcngc (conserved a.a. IDAA)
ggtCODEHOPrw-k	CAATTCTAATTTCATCAGGTAGCcaytgcatrtg (conserved a.a. HMQW)
ggtCODEHOPfw-g	ATCCATATTATGGCTGAAGCTatgmgncargc (conserved a.a. MRQA)
ggtCODEHOPrw-g	ACGCTTCTATCAGCATAAGCTtgnckcatngc (conserved a.a. MRQA)
ggt_seqCODEHOPfw-b	GATGAAGGCGGGAATGCT
ggt_seqCODEHOPrw-k	CAATTCTAATTTCATCAGGTAGC
ggt_seqCODEHOPfw-g	ATCCATATTATGGCTGAAGC
ggt_seqCODEHOPrw-g	ACGCTTCTATCAGCATAAGCT
1341fw	AAGTCGCACCAAAGGCAGCTAGTC
1341rw	ATCGCTTGCTGTCGCCCTATTTGTC
1828fw	AAGGTGGGGATACACTCG
1828rw	GGTGATTCTACCGCTTTTGC
1341fw-RFLP	GCAAAAAGAAGGCGAGAGTG
1341rw-RFLP	TGAAAGCGTGGAGATTCTAC
HRAG1341pBADKpnI	AGCAGGTACCGCTATTATCATATCTAAAACCGCTAG
HRAG1341FwNdeI	ACGCCATATGATGCTATTATCATATCTAAAACCGCTAG
HRAG1341RwHindIII	CCCAAGCTTTTATATCTTCCTTCTAGGGTCTAATGTG
HRAG1828pBADKpnI	AGCAGGTACCGACAGGGTTTTTAAGCGTGAGTG
HRAG1828FwNheI	CTAGCTAGCATGACAGGGTTTTTAAGCGTGAG
HRAG1828RwHindIII	CCCAAGCTTTTAAAACTCTTTTCTTGGATCATTTACTC
HRAG1828up5PstI	ATTCTGCAGATGACAGGGTTTTTAAGCGTGAG
HRAG1828up3XbaI	ATTCTAGAGTGATTACCCTCTTTGTGATTTATAGGTTGTCC
HRAG1828dw5KpnI	ATGGTACCGCAAGTTATGGTGCGATAGCAGAGGTTGAAG
HRAG1828dw3EcoRI	ATGAATTCTTAAAACTCTTTTCTTGGATCATTTACTC
U1catF2	ATTCTAGACGGCGGTGTTCCTTTCCAAG
U1catR	ATGGTACCCGCCCTTTAGTTCCTAAAGGG
up1828	CGTCAGTTAAATTACTTGCAGCC
dw1828	CTTAAAAGGGGAGAGTTTATTTACCTG
CatR	CCCTTATCGATTCAAGTGCATCATG
CatL	TAGTGGTCGAAATACTCTTTTCGTG

### Frequency of *bgh1* and *bgh2* genes in *H. bilis* strains

To determine the frequency of *bgh1* and *bgh2* in *H. bilis*, 33 *H. bilis* strains from our collection [38], including *H. bilis* type strain ATCC 51630^T^, *H. bilis* ATCC 49314 and *H. bilis* ATCC 49320, were subjected to a specific *BclI* RFLP-PCR for *bgh1* and a specific PCR for *bgh2*. All strains were positive for the presence of both genes. Furthermore, *bgh1* orthologues of *H. bilis* type strain ATCC 51630^T^ and three canine strains [38] were sequenced and uncorrected distance matrices were constructed on the basis of both nucleotide and amino acid sequences. The overall nucleotide sequence identity observed varied from 94.7% to 97.3%, while the amino acid identity ranged from 97.5% to 100%.

### Transcription of *bgh1* and *bgh2* by *H. bilis* CCUG 23435

The transcription of both *bgh1* and *bgh2* was evaluated in *H. bilis* CCUG 23435 at different time points. Bacterial growth was monitored by measuring OD_600_ up to 24 h, corresponding approximately to the end of the exponential phase. At 8, 12 and 24 h both genes were transcribed (data not shown).

### Expression, purification and autoprocessing of recombinant Bgh1 and Bgh2 proteins

**Figure 4:**
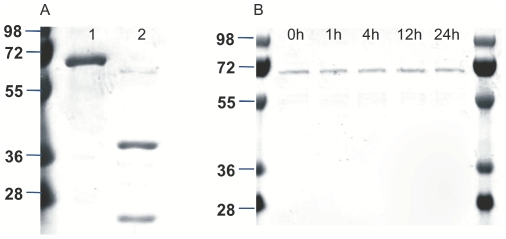
Purity and autoprocessing of recombinant Bgh1 and Bgh2. (A) SDS-page of the purified proteins after gel filtration: Bgh1 (Lane 1) and fully autoprocessed Bgh2 (Lane 2); (B) time-line for autoprocessing of Bgh1.

To further analyse the biochemical characteristics of both *H. bilis* γGT paralogues, we expressed recombinant His-tagged Bgh1 and Bgh2 proteins in *E. coli*. Bgh2 was expressed without the signal peptide, while for Bgh1 the entire sequence was used. Recombinant expression of Bgh2 resulted in a soluble protein that was directly purified by Ni-affinity chromatography. By contrast, Bgh1 was expressed as an insoluble protein of ∼70 KDa and was purified after refolding. The purity of the proteins on SDS-PAGE was >90% (Figure 4a). Bgh2 showed a catalytic activity for the substrate analogue L-γ-glutamyl-p-nitroanilide (gGpNA) and was synthesized as a pro-form of ∼60 KDa, which undergoes autocatalytic processing to generate two subunits of ∼40 and ∼20 KDa (Figure 4a). By contrast, after solubilisation and refolding, recombinant Bgh1 showed no significant maturation after 24 h of incubation at 37°C and no activity for gGpNA (Figure 4b). To estimate the secondary structure of Bgh1 and to verify the absence of a random folding, a Circular Dichroism (CD) spectrum of the recombinant protein was calculated. The spectrum minimum calculated for recombinant Bgh1 was 206 nm, indicating the absence of random coil. The secondary structure of Bgh1 was predicted by K2D3 [39] to contain 30.23% of alfa helix and 16.82% of beta sheet. The CD spectrum for Bgh1resulted similar to the spectrum predicted by K2D3 and to those for proper folded proteins, e.g. lysozyme [40], indicating that recombinant Bgh1 has a defined secondary structure (Figure S1).

### Autoprocessing of Bgh1 and Bgh2 in *H. bilis*

**Figure 5:**
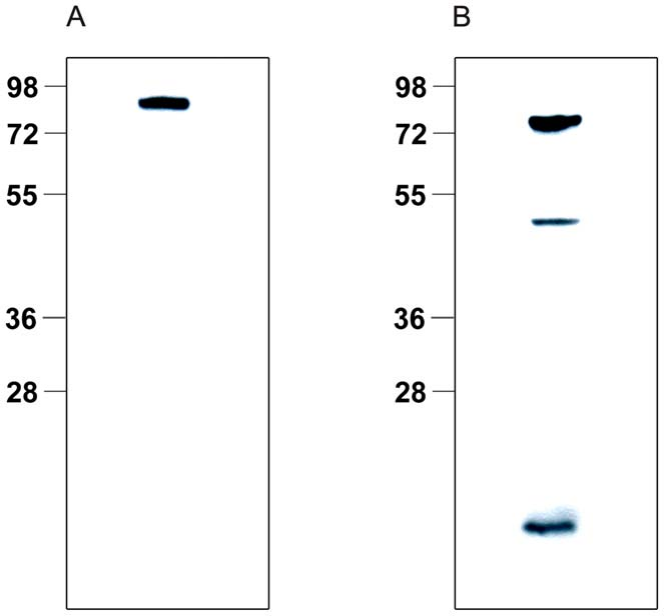
Western Blot on whole *H. bilis* lysate using specific antisera against Bgh1 and Bgh2. Western blot of whole *H. bilis* lysate using antisera against (A) Bgh1 and (B) Bgh2.

In order to evaluate the maturation of both Bgh1 and Bgh2 in *H. bilis*, antisera against both recombinant proteins were produced in mice and western blot analysis on *H. bilis* whole lysate was performed. The antisera showed high specificity for the corresponding *H. bilis* γGT paralogue in ELISA test at 1∶900 dilution selected for the subsequent western blots (data not shown). The western blot performed on *H. bilis* whole lysate using the antiserum against Bgh1 clearly showed a single band without indication of autoprocessing (Figure 5a). On the contrary, the western blot carried out using antiserum against Bgh2 revealed the expression of the pro-form and its corresponding two subunits (Figure 5b). These results confirmed that both proteins are expressed in *H. bilis* and that only Bgh2 undergoes autocatalytic processing.

### Complementation of *E. coli* Δggt

To evaluate the capability of *H. bilis* γGT paralogues to functionally complement an *E. coli* γGT-deficient strain, Bgh1 or Bgh2 (carried on plasmid pMRg3 and pMRg5, respectively) were introduced in *E. coli* CY128 [41]. *E. coli* DH5α grown in LB overnight at 25°C was used as positive control in γGT assay. Only Bgh2 was found to successfully complement *E. coli* CY128, while Bgh1 was unable to restore γGT activity (data not shown). The induction of pMRg3 in *E. coli* CY128 resulted in a clear inhibition of growth. This finding was consistent with formation of inclusion bodies, as a consequence of the overexpression of Bgh1. However, when the protein was expressed overnight at a lower temperature (15°C) with low amount of inducer (0.002% instead of 0.2%), there was no inhibition of the growth rate of *E. coli*, suggesting a low accumulation of insoluble proteins. The successful expression of Bgh1 under improved conditions was confirmed by SDS-PAGE (data not shown). Nevertheless, Bgh1 was not able to complement *E. coli* Δ*ggt*, while Bgh2, expressed under the same conditions, restored γGT activity in the mutant strain.

### Construction of a *H. bilis* mutant deficient in γGT activity

To confirm that Bgh2 is responsible of the γGT activity in *H. bilis*, a mutant of *bgh2* was constructed in CCUG 23435 strain. The *bgh2* gene was disrupted by insertion of a chloramphenicol cassette between positions 1182 and 1246, in order to delete the threonine-threonine catalytic dyad. The successful insertion of the choramphenicol resistance cassette and the deletion of *bgh2* were confirmed by PCR. RT-PCR and western blot analysis showed the expression of *bgh1* in both wild-type and mutant strains (data not shown), indicating that the replacement of *bgh2* had no effect on the expression of the *bgh1* paralogue gene. Since complementation approaches are currently unavailable for *H. bilis*, another independent Δ*bgh2* mutant was used as a control for secondary mutations. Neither of the *H. bilis* mutants were able to hydrolyse the substrate analogue gGpNA. Moreover, in the supernatant of *H. bilis* wild-type γGT activity was determined with a gGpNA turnover of 0.48 µM/min at a concentration of 50 µg/mL of total protein. No activity was detected in the supernatant of the mutant strain MR9 (Figure S2). These results indicate that only *bgh2* encodes a functional γGT in *H. bilis* CCUG 23435 (Hb-γGT). The mutants grew normally *in vitro*, as described for *H. pylori* γGT-deficient strains [9], indicating that γGT is not essential for survival and growth of both *H. pylori* and *H. bilis in vitro*.

### Biochemical characterization of recombinant Bgh2 (Hb-γGT) and comparison with *H. pylori* γGT (Hp-γGT)

**Figure 6:**
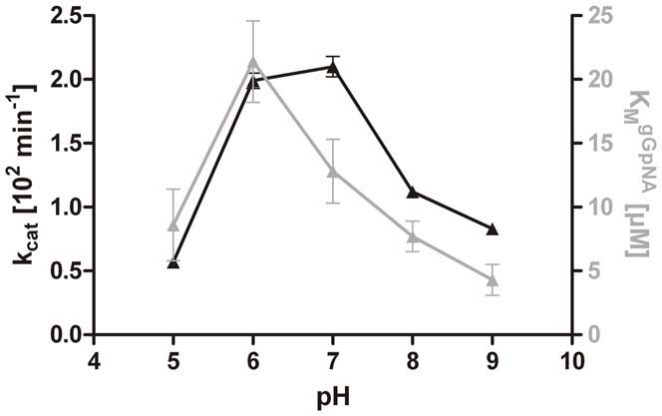
Comparison of the kinetic constants for *Helicobacter bilis* γGT (Hb-γGT) at different pH. Error bars in the graphs were calculated as SEM. The analysis was performed using Prism4 v4.03 (GraphPad Software, San Diego, CA USA). At indicated pH values, kinetic constants (k_cat_ in black and KM in grey) for the hydrolysis of L-γ-glutamyl-p-nitroanilide (gGpNA) were determined.

The kinetic parameters of recombinant Bgh2 (Hb-γGT) were measured. The initial experiments were performed at pH 8.0. The K_M_ was determined by titration of the gGpNA concentrations between 1 and 1000 µM. The processed Hb-γGT had a K_M_ of 7.7±1.2 µM and a k_cat_ of 1.12±0.03•10^2^/sec. Compared with K_M_ and k_cat_ values calculated for Hp-γGT (K_M_ = 9.8±1.5 µM and k_cat_ = 17.7±0.5•10^2^/sec), the apparent K_M_ value for Hb-γGT was 20% lower, while the k_cat_ value was >10-fold reduced (Table 2). The pH dependence of the Hb-γGT activity was analysed and compared to Hp-γGT (Figure 6). The pH profile of Hp-γGT activity was similar to reported data [30] (data not shown), whereas the activity optimum of Hb-γGT was shifted to a more acidic pH between 6.0 and 7.0. By contrast, the K_M_ was increased more than 2-fold at this pH range. Initial experiments for comparing the substrate specificity of Hp-γGT and Hb-γGT for glutathione, D-glutamine, L-glutamine and L-glutamic acid were performed by a competition assay. A better binding affinity to the active γGT and therefore a competitive inhibition of the gGpNA-reaction would be expected for its native substrates. As previously described [9], [12], Hp-γGT substrate inhibition was observed for both by glutathione and L-glutamine, whereas Hb-γGT was only inhibited by L-glutamine (Figure S3).

**Table 2:** Comparison of the kinetic constants for *Helicobacter bilis* γGT (Hb-γGT) and *Helicobacter pylori* γGT (Hp-γGT).

	*k_cat_ [10^2^ min^−1^]*	*K_M_^gGpNA^ [*µ*M]*
**Hp-γGT**	17.7±0.5	9.8±1.5
**Hb-γGT**	1.12±0.03	7.7±1.2

### Inhibition of human T-cell and AGS cell proliferation

**Figure 7:**
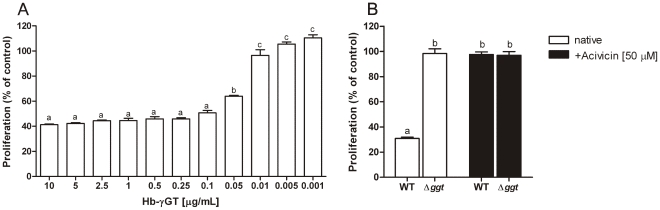
Inhibitory effect on T-cell proliferation (Jurkats) by *Helicobacter bilis* γGT (Hb-γGT). Error bars in the graphs were calculated as SEM. The analysis was performed using Prism4 v4.03 (GraphPad Software). Different letters on the bars indicate significant differences at P<0.05. (A) Inhibitory effect with various amounts of recombinant Hb-γGT (Bgh2) protein; statistical analysis was performed using one-way ANOVA, followed by the Bonferroni test. (B) Inhibitory effect of the culture supernatant of *H. bilis* wild-type CCUG 23435 (WT) and *H. bilis* Δ*ggt* MR9 (Δ*ggt*) with or without pre-treatment with Acivicin (Sigma-Aldrich); statistical analysis was performed using unpaired *t*-test.

To evaluate biological properties of recombinant Hb-γGT and compare these with effects already observed for Hp-γGT, the ability of Hb-γGT to inhibit human T-cell and AGS cell proliferation was assessed. Recombinant Hb-γGT efficiently inhibited the proliferation of Jurkat cells in a dose-dependent manner (Figure 7a). Concentrations of Hb-γGT as low as 0.1 µg/mL induced inhibition of T-cell proliferation. Comparison of the inhibitory potential of supernatants of wild-type and Δ*ggt* strains confirmed the results obtained with the recombinant proteins. The supernatants of *H. bilis* wild type reduced T-cell proliferation up to 60%, while the supernatant of Δ*ggt* strain had no significant inhibitory activity. When Jurkat cells were infected with acivicin pre-treated supernatant of *H. bilis* wild type, no significant reduction in proliferation of T-cells was observed, indicating that only Hb-γGT is responsible for the inhibition of T-cell proliferation in our system (Figure 7b).

**Figure 8:**
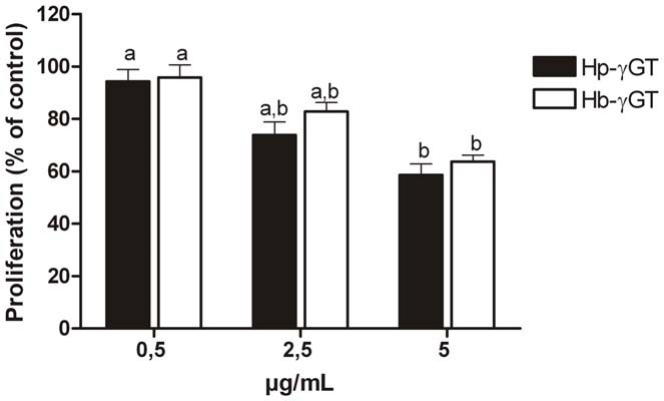
Inhibitory effect on AGS proliferation by *Helicobacter bilis* γGT (Hb-γGT). Error bars in the graphs were calculated as SEM. The analysis was performed using Prism4 v4.03 (GraphPad Software). Statistical analysis was performed using one-way ANOVA, followed by the Bonferroni test. Different letters on the bars indicate significant differences at P<0.05.

Moreover, both recombinant Hb-γGT and Hp-γGT showed inhibitory effect on AGS cell proliferation in a dose-dependent manner as well, although the minimum concentration of protein required was 25 time higher (2.5 µg/mL) compared to that able to inhibit the proliferation of human T-cells (Figure 8). No statistically significant differences were observed between the two proteins.

### Apoptosis in AGS cell line

The ability of recombinant Hp-γGT and Hb-γGT to induce apoptosis in AGS cells was evaluated using AnnexinV-Propidium Iodide double staining and FACS analysis. After 24 h of incubation, concentrations of up to 5 µg/mL of both recombinant proteins did not induce apoptosis in AGS cells (Figure S4). The average of percentage of apoptosis observed after treatment with Hp-γGT and Hb-γGT was 6.9±1.2% and 6.8±0.9%, respectively. Those values were not statistically different from the percentage of apoptosis measured in the untreated cell (7.3±1.9).

## Discussion

Two *Helicobacter* γGT genes, identified in the shotgun genome sequence of the human strain *H. bilis* ATCC 43879 on the basis of sequence homology, were subjected to functional analysis to confirm their annotations. Although the two genes *bgh1* and *bgh2* (*H. bilis ggt* homologue 1 and 2) showed significant homology and both are classified as T03.01 peptidases in MEROPS, the evaluation of conserved amino acid residues indicated a marked difference between these two paralogues, especially in the substrate-binding pocket. In fact, all critical functional residues are present in Bgh2, as well as in all the other *Helicobacter* γGTs, but not in Bgh1. The amino acid substitutions observed in Bgh1 could potentially affect the enzyme maturation, catalytic activity and substrate specificity, suggesting different biochemical properties. Moreover, the absence of a signal peptide in Bgh1, in contrast to observations for Bgh2 and for the other *Helicobacter* γGTs, suggests a different sub-cellular localization of this protein.

We first evaluated the phylogeny of *Helicobacter* γGTs in relation with T03 proteases of other bacterial species. The Minimum Evolution tree clearly showed that both *H. bilis* T03.01 peptidase paralogues were phylogenetically associated with other ε-proteobacteria γGTs. Nevertheless, Bgh1 formed an independent branch, indicating a different evolution. Moreover, among *Helicobacter* species, only *H. trogontum* showed two distinct γGT paralogues, as observed in *H. bilis*. In addition, the data suggested that both genes are not restricted to *H. bilis* ATCC 43879, but are common features among *H. bilis* genomospecies and universally shared by all members of the taxon.

A more detailed phylogenetic analysis further revealed that the two subunits of γGTs have followed different evolutionary paths. The tree based on the C-terminal part resembled the topology of the tree of 16S rRNA and other housekeeping genes and includes *H. bilis* in the same branch as other enterohepatic *Helicobacter* spp. [38]. On the contrary the phylogenetic analysis of the N-terminal part separates the γGTs of *H. bilis* and related species (*H. aurati*, *H. muridarum* and *H. trogontum*) from others enterohepatic *Helicobacter* species. On the basis of our phylogenetic analysis we can hypothesise that differing phylogeny of the subunits reflects a possible divergence of substrate specificity between the γGTs of *H. bilis* and related species and the other *Helicobacter* or *Campylobacter* γGTs. More studies are needed to verify this hypothesis.

In the trees based on the sequences of both subunits, the position of the clade including the orthologues of *bgh1* indicated that the paralogues potentially evolved by gene duplication from a common ancestral sequence probably before the speciation event of *H. bilis* and related species. The hypothesis of gene duplication is well supported also by the low Ka∶Ks ratio between the two paralogues (0,175), indicating that the two genes are under strong purifying selection [42].

Both sequence and phylogenetic analyses reveal clear differences between the two *H. bilis* T03.01 peptidases paralogues. To compare their biochemical properties, both genes were overexpressed in *E. coli* and the recombinant proteins were purified by affinity chromatography. The overexpression of Bgh1 resulted in formation of inclusion bodies due to the accumulation of an insoluble protein of ∼70 KDa. After solubilisation and refolding, recombinant Bgh1 showed no significant maturation into two subunits and, subsequently, no γGT activity. In contrast, recombinant Bgh2 was expressed in soluble form as a pro-enzyme of ∼60 KDa, which undergoes autocatalytic processing to generate two subunits of ∼40 and ∼20 KDa and showed γGT activity. No clear molecular evidence explains the absence of an autocatalytic process for Bgh1 since all of the amino acid residues critical for Hp-γGT [2], [43] and Ec-γGT [44], [45] autoprocessing are also conserved in Bgh1. We, therefore, cannot exclude that the absence of maturation could be due to misfolding *in vitro*. However, the CD spectrum of purified Bgh1 showed that the protein has a defined set of secondary structures indicating a non-random folding of the recombinant protein. Moreover, Bgh1 was unable to restore γGT activity after complementation of *E. coli* Δ*ggt* mutant. Furthermore, the *H. bilis* strain in which *bgh2* was disrupted but still able to transcribe *bgh1* resulted in deficient γGT activity. This data clearly indicate *bgh1* does not encode a functional *H. bilis* γGT. As reported previously, the single amino acid substitution D433N converts Ec-γGT to a class IV cephalosporin acylase [46]. Since in Bgh1 the same substitution is present, we decided to check the potential cephalosporin acylase activity of *H. bilis* CCUG 23435, which expresses both γGT paralogues, as previously described for *E. coli* [46]. *H. bilis* did not show any cephalosporin acylase activity (data not shown), indicating that glutaryl-7-aminocephalosporanic acid is unlikely to be a substrate for Bgh1. To elucidate whether the lack of enzyme activity of Bgh1 is caused by the absence of autocatalytic maturation, western blot analysis on *H. bilis* whole lysate using specific antisera for both γGT paralogues were performed. The results showed that *H. bilis* expresses Bgh1 which does not undergo autocatalytic processing, confirming the results obtained with the recombinant protein. However, further studies are needed to determinate potential substrates for Bgh1 and to investigate its role in the metabolism of *H. bilis*.

Analysis of the recombinant protein, western blot on *H. bilis* whole lysate, complementation of *E. coli* Δ*ggt* and deletion of the gene in *H. bilis* CCUG 23435 clearly showed that only *bgh2* encodes the functional *H. bilis* γGT. Hb-γGT exhibited similar affinity to the substrate analogue gGpNA at pH 8.0 compared with that of Hp-γGT; however, it was significantly less proficient, having more than 10-fold less k_cat_. Moreover, in contrast to observations for Hp-γGT [30], the affinity of Hb-γGT to gGpNA varied in relation to the pH, being stronger at acidic and alkaline conditions and decreasing significantly at pH 6.0 and 7.0. In addition, Hb-γGT showed the highest activity at pH 6.0, which is lower compared to previous reports for *H. pylori* and other bacterial γGTs [30]. The results of the competition assay suggested that Hb-γGT and Hp-γGT have similar substrate specificity, utilizing not only the substrate analogue gGpNA but also L-glutamine. Nevertheless, Hb-γGT differs from Hp-γGT in its inability to bind glutathione as substrate. Therefore, we can speculate that *H. bilis* γGT lost its ability to interfere the host redox system. However, the enzyme enables the bacterial cells to use extracellular glutamine as the source of glutamate. This hypothesis is supported by the presence in the *H. bilis* genome of a putative glutamate transporter gene (HRAG_00091; *gltS*) showing ∼60% of amino acid identity with *H. pylori* GltS homologue [47]. Glutamate transporters *gltS* as well as *ggt* genes are missing in the EHS *H. hepaticus* genomes [47]. Both *H. hepaticus* and *H. bilis* are urease-positive *Helicobacter* spp., commensal bacterial species and/or opportunistic pathogens of mice, sharing the same niches in the lower gastrointestinal tract and causing similar diseases [48]. However, *H. bilis*, but not *H. hepaticus*, was also isolated from the stomach of different hosts [19]. Therefore, the expression of γGT activity by *Helicobacter* spp. does not appear to be essential for colonization of the lower gastrointestinal tract of mice, but it could provide metabolic advantages in colonization capability of other tissues or in the adaptation of different hosts.

Although the importance of γGT for physiology and pathobiology of mammalian tissues has been known for many years, its function in prokaryotic metabolism and its role in interaction with the host have been studied for only a few bacterial species [6], [9], [10], [12], [47], [49]–[51]. Recently it has been showed that γGT has an important role in the interaction between *H. pylori* and the host by inhibiting T-cell proliferation [9], inducing apoptosis in gastric cells [10] and contributing to *H. pylori*–mediated H_2_O_2_ generation [8]. Here, we showed that *H. bilis* γGT inhibited T-cell proliferation similar to what was observed for *H. pylori* [9], [10]; the inhibitory effect of T-cell proliferation was completely abolished in the γGT-deficient mutants of *H. bilis*. Our data demonstrate that this activity is not limited to *H. pylori* but is conserved among the genus, confirming the potential immune suppressive role of secreted *Helicobacter* γGTs. The conserved function of this enzyme among *Helicobacter* spp. with different niches and host specificity opens a new scenario on the role of γGT in the pathogenesis of *Helicobacter* associated diseases.

We detected AGS cell proliferation inhibition after treatment with both recombinant γGTs, as observed for T-cells, but only when a high concentration was used (2.5 µg/mL). However, in contrast to what was previously described [10], we showed that the suppressive influence of both Hb-γGT and Hp-γGT on AGS cells was mediated by an apoptosis-independent mechanism. The discrepancy between our results and those of Shibayama and colleagues [10] could attribute to different methodologies applied, in particular to the use of serum starvation by these authors. An increase of apoptosis was verified in AGS cells treated with both Hp-γGT and Hb-γGT under the same conditions used by these authors (data not shown), apparently confirming the apoptosis inducing potential of these enzymes. However, if this phenomenon is only detectable upon stress conditions (i.e. serum starvation), we cannot exclude that the observed apoptosis is a consequence of a nonspecific activity of γGT. Further studies are needed to establish the real role of γGT in the apoptosis of epithelial cells.

## Methods

### Bacterial strains, cell line, culture condition and DNA extraction

The bacterial strains and plasmids used in this study are listed in Table 3 and 4. All *Helicobacter* strains were routinely grown on HP medium (LabM Limited, Lancashire, UK) containing 5% bovine blood at 37°C in a microaerobic atmosphere (10% CO_2_; 5% O_2_) (Thermo Forma, Series II Water Jacketed Incubator; Thermo Fisher Scientific, Waltham, MA USA). *E. coli* strains were cultivated on Luria-Bertani (LB) agar or broth supplemented with 100 mg/L of ampicillin, 15 mg/L of kanamycin or 20 mg/L of chloramphenicol when needed. Jurkat T-cells (DSMZ, ACC 282, Deutsche Sammlung von Mikroorganismen und Zellkulturen GmbH, Braunschweig, Germany) were cultured in RPMI 1640 (Invitrogen Corporation, Carlsbad, CA, USA) with 10% of foetal bovine serum (FBS; Gibco, Invitrogen). AGS cells (CLS, 300408; Cell Lines Service, Eppelheim, Germany) were grown in Dulbecco's Modified Eagle's growth medium (DMEM high glucose, 11971, Invitrogen) supplemented with 10% FBS and 200 U/mL of penicillin and 0.2 mg/mL of streptomycin (Pen Strep, Gibco, Invitrogen). Cell lines were incubated at 37°C with 5% CO_2_. Bacterial DNA extraction was performed as described earlier [19].

**Table 3:** *Helicobacter* species used in this study.

Species	Strain designation and genotype	Hosts	Reference
	[*ggt* locus tag or accession number and/or *bgh1* homologue accession number]		
**Gastric ** ***Helicobacter*** ** spp.**			
*H. acinonychis*	Sheeba [*ggt*, Hac_0598]	Cheetah; Big Felines	[60]
*H. bizzozeronii*	CIII-1 [*ggt*, HBZC1_08080]	Dog; Human	[61]
*H. felis*	ATCC 49179 [*ggt*, Hfelis_06880]	Cat	[62]
*H. mustelae*	12198 [*ggt*, HMU08020]	Ferret	[63]
*H. pylori*	26695 [*ggt*, HP1118]	Human	[64]
*H. salomonis*	O6A [*ggt*, EMBL FR821684]	Dog	[38]
*H. suis*	HS1 [*ggt*, HSUHS1_0265]	Pig	[65]
**Enterohepatic ** ***Helicobacter*** ** spp.**			
*H. aurati*	ATCC BAA-1^T^ [*ggt* EMBL FR821682]	Hamster	[38]
*H. bilis*	CCUG 23435 ( = ATCC 43879)[*ggt*, HRAG_01828; *bgh1*, HRAG_01341]	Mouse; Human; Dog; Cat; Pig; Sheep	[19]
	MR9 ( = CCUG 23435 Δ*bgh2*)		
	ATCC 51630^T^ [*bgh1* homolog EMBL FR821680]		
	ATCC 49314		
	ATCC 49320		
	KO220 [*bgh1* homologue EMBL FR821686]		
	KO214 [*bgh1* homologue EMBL FR821687]		
	KO794 [*bgh1* homologue EMBL FR821688]		
	FL56 [*ggt* EMBL FR821679] [*bgh1* homologue EMBL FR821676]		
*H. canis*	NCTC 12739^T^ [*ggt* EMBL FR821681]	Dog; Cat; Human	[66]
*H. muridarum*	CCUG 29262^T^ ( = ATCC 49282^T^) [*ggt* EMBL FR821683]	Mouse	[38]
*H. trogontum*	ATCC 700114^T^ [*ggt* EMBL FR821678] [*bgh1* homologue EMBL FR821677]	Rat; Mouse; Pig	[38]

**Table 4:** *E. coli* strains and plasmids used in this study.

Strains or plasmids	Relevant characteristics or genotype	Reference
***Escherichia coli*** ** strains**		
DH5α	F^−^ endA1 glnV44 thi-1 recA1 relA1 gyrA96 deoR nupG Φ80d*lacZ*ΔM15 Δ(*lacZYA-argF*)U169, hsdR17(r_K_^−^ m_K_^+^), λ–	[67]
BL21 Rosetta 2	F^−^ *ompT hsdS*_B_(r_B_^−^ m_B_^−^) *gal dcm* (DE3) pLysSRARE2 (Cam^R^)	Novagen
JM109	endA1 glnV44 thi-1 relA1 gyrA96 recA1 mcrB^+^ Δ(lac-proAB) e14- [F′ traD36 proAB^+^ lacI^q^ lacZΔM15] hsdR17(r_K_^−^m_K_^+^)	Promega
CY128	DH5α but Δ*ggt* Δ*ampC*	[41]
**Plasmids**		
pGEM®-T		Promega
pet28b(+)		Novagen
pBAD24	pMB1/M13 replicon, Ap^+^, AraC^+^, terminator (*rrnB*), promoter ParaBAD	[57]
pUC119	pMB1/M13 replicon, Ap^+^, *lacZ*α,	[68]
pUOA14	pMB1/M13 replicon, pIP1445 replicon, *lacZ*′, Cm^+^, Ap^+^, Km^+^	[58]
pMRg1	pet28b(+), His_6_-Bgp1^+^	this study
pMRg2	pet28b(+), His_6_-Bgp2^+^	this study
pMRg3	pBAD24, Bgp1^+^	this study
pMRg5	pBAD24, Bgp2^+^	this study
pMRg9	pUC119, *bgp2*::*cam* ^+^	this study

### PCR, cloning and sequencing

Consensus DEgenerate Hybrid Oligonucleotide Primers [iCODEHOP [52]] were designed on the basis of highly conserved amino acid sequences of available *Helicobacter* and *C. jejuni* γGTs. Standard PCR was done in a 50 µL reaction mixture containing 50 ng of DNA template, 25 pmol of each primer and 1.25 U of DyNAzyme™ (Finnzymes Oy, Espoo, Finland). The 5′ consensus clamp region contains the sequencing primers used for direct sequencing of the PCR products. If secondary peaks or multiple sequences were present, the PCR products were inserted into pGEM®-T and sequenced on both strands using M13 primers. The area corresponding to nucleotides 702–1569 (amino acids 234–523) of *bgh1* was used to design specific primers for RFLP-PCR protocol. The PCR was done in a 50 µL reaction mixture, as described above, and then the products were digested by *BclI* (NEB, New England Biolabs, Ipswich, MA, USA). *BclI* specifically cuts the PCR fragment in two pieces of 606 and 260 bp. EMBL accession numbers are listed in Table 3. All oligonucleotides used in this study are listed in Table 1.

### Phylogenetic analysis

Phylogenetic analyses of γGT amino acid sequences were conducted in MEGA4 [53] using the Minimum Evolution (ME) method. The amino acid sequences were aligned using MAFFT [54]. The evolutionary distances were computed using the Dayhoff matrix-based method, and the ME tree was searched using the Close-Neighbor-Interchange (CNI) algorithm (level 1). All positions containing alignment gaps and missing data were eliminated in pairwise sequence comparisons [53]. A multi-alignment of *ggt* sequences of *Helicobacter* spp and *C. jejuni* was built in MEGA4 on the basis of the amino acid sequence alignment. Maximum likelihood (ML) trees were constructed using PHYML [55] with a nucleotide evolution model selected by FindModel (http://www.hiv.lanl.gov/). Trees were visualized by iTOL [56]. Non-synonymous/synonymous substitution (Ka∶Ks) was evaluated by sliding window analysis (window size of 50 bp) using SWAKK [37].

### Expression of Bgh1 and Bgh2 and purification of recombinant proteins

Primers pairs were designed to amplify complete sequences of *bgh1* and *bgh2* genes using Phusion® High-Fidelity DNA Polymerase (Finnzymes). Bgh1 and Bgh2 were expressed as 6×His-tagged proteins by inserting the genes respectively in *NdeI* and *HindIII* or *NheI* and *HindIII* restriction sites of pet28b+ vector (Novagen®, Merck KGaA, Darmstadt, Germany). The 6×His-tag was fused in the N-terminal part of the proteins. The resulting expression constructs pMRg1 and pMRg2 were sequenced and confirmed to be identical to *bgh1* and *bgh2*, respectively. Both expression constructs were used to transform *E. coli* Rosetta 2 (Novagen®). Expression of recombinant protein was induced with 0.5 mM IPTG for 4 h at 27°C. After centrifugation, pellets were solubilized in ice-cold binding buffer (50 mM Tris-HCl, 500 mM NaCl, 10 mM imidazole, pH 7.4) containing protease inhibitors and then lysed by subsequent sonication (5 times for 30 s) on ice. The supernatant was loaded on the HisTrap™ column (GE Healthcare Biosciences, Pittsburgh, PA, USA) at 1 mL/min at 4°C, and bound protein was eluted with an imidazole-gradient (0–100 mM imidazole) using the elution buffer (50 mM Tris-HCl, 500 mM NaCl, 1000 mM imidazole, pH 7.4). Eluates were collected and then tested for purity by SDS-PAGE. Since purification of a soluble form of Bgh1 was not possible, the sonicated cells of *E. coli* (4 times for 1 min) were homogenized, solubilized in 6 M Gua-HCl, 1 mM DTT, 10% Glucose pH 8.5 and stirred for 1 h at 4°C. Following centrifugation, the supernatant containing the unfolded protein was loaded on a HisTrapHP™ column (GE Healthcare) at 4°C. The bound protein was refolded with a gradient of 100 mM HEPES, 5% Glucose, 250 mM NaCl, 10 mM MgCl_2_, 1 mM DTT pH 8.5 (0–100% of HEPES buffer). After the refolding, the column was washed extensively with 5 column volumes of HEPES buffer, and bound protein was eluted with an imidazole-gradient (0–1000 mM) using HEPES buffer with 1000 mM imidazole. Eluate was collected and then tested for purity by SDS-PAGE. For further purification, fractions with the recombinant proteins from Ni-Sepharose affinity chromatography were pooled, dialysed overnight against 100 nM HEPES, 140 mM NaCl, 2.5% Sucrose pH 7.0, at 4°C and processed to the second and final purification step. The dialysed samples were loaded on a Superdex™75 column (GE Healthcare) and equilibrated with 100 nM HEPES, 140 mM NaCl, 2.5% Sucrose pH 7.0. All fractions collected were analysed by SDS-PAGE for the presence of recombinant proteins. Fractions containing the protein were pooled, aliquoted and stored at −80°C until further use.

### Circular Dichroism (CD) spectrum of the recombinant Bgh1

To estimate the secondary structure of Bgh1 a CD spectrum of the recombinant protein was calculated. The measurements were performed on a JASCO J-810 spectropolarimeter equipped (Jasco Labor- und-Datentechnik GmbH, Gross-Umstadt, Germany) using a 0.2-mm-pathlength quartz-glass cuvette 1-mm. CD spectra were recorded with 50 µl of the sample at final concentration of 15 µM in 20 mM KH_2_PO_4_ pH 7.5, 50 mM K_2_SO_4_. All CD measurements were corrected by subtracting the buffer spectrum. The following parameters were used: data pitch, 0.1 nm; continuous scanning mode at 100 nm/min; response, 4 sec; band width, 1 nm; accumulation, 16. Smoothing was made by the method Means-Movement. Data were collected using Spectra manager v1.54.03 (Jasco).

### Immunization of mice and Western-Blot on whole *H. bilis* lysate

Balb/c mice (3 mice per group) were immunized i.p. with 30 µg of antigen and 10 µg Cholera toxin (CT) (Sigma-Aldrich) as adjuvant at days 0, 7 and 14 (control group with CT only). Sera were taken 21 days after the last immunization and stored at −20°C. Animal experiments were performed according to the guidelines of the Bavarian Ministry of Animal Wealth Fare. For evaluation of Bgh-specificity, ELISA plates were coated with 2 µg/mL recombinant Bgh1 and Bgh2 proteins in PBS pH 7.4 overnight at 4°C. After washing (4×0.01% Tween-20 in PBS) and blocking (1% BSA in PBS), different dilutions of sera (in blocking buffer) were incubated for 1 h at 37°C. HRP-conjugated anti-mouse IgG antibody (1∶3000) (Promega Corporation, Fitchburg, WI, USA) was incubated (1 h at 37°C) and detected with ELISA Opt EIATM (BD, Becton, Dickinson and Co., NJ, USA) according to the manufacturer's instructions.

For the western blot, *H. bilis* overnight culture was resuspended in 1 mL BHI (BD). Following lysis (sonication 4×30 sec), the protein-concentration in cleared lysates was determined by BCA-Assay and adjusted to 1 mg/mL. Per lane 5 µg of total protein were loaded. After blocking with 5% BSA in Tris-buffered saline (TBS), the Whatman Protran® Nitrocellulose membranes (GE Healthcare) were incubated for 1 h at RT with the sera of the immunized mice (1∶800 diluted in TBS). Following washing, HRP-conjugated secondary anti-mouse IgG antibody (Promega) (diluted 1∶3000 in TBS) was added and detected by the acridan-based ECL-system (Thermo Fisher Scientific).

### Complementation of *E. coli* Δ*ggt*

*bgh1* and *bgh2* were amplified as described above and were inserted between *Kpn*I and *Hind*III restriction sites of pBAD24 expression vector [57], resulting in pMRg3 and pMRg5, respectively. Both expression constructs were used to transform *E. coli* CY128 Δ*ggt*. The recombinant strains were grown in LB medium with ampicillin at 37°C to OD_600_≈0.4, and the expression of the corresponding gene was induced with 0.2% L-arabinose for 4 h at 25°C. The basal expression of pMRg3 and pMRg5 vectors was inhibited by using 0.2% of glucose instead of L-arabinose. Expressions were verified by SDS-PAGE electrophoresis and Coomassie Blue stain. A total of 10^9^ cells were harvested by centrifugation and stored at −70°C before the γGT assay.

### Construction of *H. bilis* isogenic mutant

Chromosomal inactivation of *bgh2* was performed in *H. bilis* CCUG 23435. Deletion was introduced by allelic exchange using vector pUC119 in which ∼1100 bp of the 5′ –end and ∼500 bp of the 3′ –end of the target gene and the chloramphenicol resistance gene from pUOA14 [58] were cloned. The resultant plasmid, pMRg9, was constructed and amplified in *E. coli* DH5α and used as a suicide plasmid in *H. bilis* CCUG 23435. *H. bilis* mutant was obtained by electroporation as described for *H. hepaticus* [59]. The mutant strain, *H. bilis* MR9 (*bgh2*::cam), was selected on an HP medium containing 5% bovine blood supplemented with chloramphenicol (20 mg/mL). The site of recombination was verified by PCR.

### RNA extraction and RT-PCR

To analyse the expression of both *bgh1* and *bgh2* during growth, *H. bilis* wild-type CCUG 23435 was cultivated in Brain Heart Infusion (BHI; BD, NJ USA) containing 20% FBS and Vitox supplement (Oxoid Ltd., Cambridge, UK) at 37°C microaerobically by continuous shaking at 150 rpm. OD_600_ was measured at 8, 16 and 24 h and the same amount of cells was treated with RNAprotect Bacteria Reagent (Qiagen GmbH, Hamburg Germany). RNA was extracted using RNeasy Mini Kit (Qiagen) and treated with TURBO DNase™ (Applied Biosystems/Ambion, Austin, TX, USA). Moreover, *H. bilis* CCUG 23435 and the mutant MR9 were cultivated in YT×2 medium (16 g/L Tryptone, 10 g/L Yeast Extract, 5 g/L NaCl) containing 10% FBS at 37°C in a microaerobic atmosphere. After 24 h RNA was extracted as described above. cDNAs were synthesized from 1 µg of total RNA using SuperScript® III Reverse Transcriptase (Invitrogen) using random hexamers (Finnzymes). RT-PCR reactions were carried out in a total volume of 50 µL as described above, using 1 µL of cDNA and 1 µL of RNA as negative control and 20 pmol of specific primers for *bgh1* and *bgh2*. Thermocycling conditions were 94°C for 2 min followed 40 cycles of 94°C for 45 s, 58°C for 45 s and 72°C for 1 min. The PCR products were sequenced using the same primers.

### T-cell and AGS proliferation assay

For measurement of the effect of *H. bilis* γGT on T-cell proliferation, Jurkat cells were seeded at 5×10^3^ per well in a complete culture medium in 96-well microtiter plates and a variable amount of protein (0.001 to 10 µg/mL) or sterile-filtrated culture supernatant (1 µg/mL total protein) was added in a total volume of 100 µl. As a control for the inhibition of T-cell proliferation, the recombinant γGT from *H. pylori* 26695 (HP-γGT) was used. The HP-γGT was produced as previously described [9]. Additionally, supernatant were preincubated with 50 µM Acivicin for 30 min at 37°C to inactivate the γGT. Cells were incubated at 37°C for 48 h and analysed for proliferation as described previously [9]. Results are expressed as the mean±SEM from three experiments.

For the measurement of the effect of recombinant *H. bilis* and *H. pylori* γGT on proliferation of AGS cells, 5×10^3^ cells per well were seeded in culture medium in a 96-well plate and treated with 0.5–5 µg/mL of protein in a total volume of 100 µl. Cells were incubated at 37°C for 48 h and analysed for proliferation as described above.

### Apoptosis in AGS cells

Apoptosis in gastric epithelial cells was determined by Fluorescence-activated cell sorting (FACS). AGS cells were seeded in a 6-well plate till they reached a confluence of 60–70%, and treated for 24 hours with 0.5–5 µg/mL of purified recombinant γGT proteins from *H. pylori* and *H. bilis* in DMEM with 10% of FBS. Untreated cells were used as a negative control and cells treated with 10 µM of Staurosporine (LC laboratories, Woburn, MA, USA) as a positive control for apoptosis. Cells were harvested with 0.25% Trypsin-EDTA (Invitrogen) and 2×10^5^ cells were washed once with PBS. The pellets were resuspended in 10 µg/mL of AnnexinV-FITC antibody (BD, Becton, Dickinson and Co., NJ USA) in FACS buffer and incubated for 20 minutes in dark at room temperature. Cell suspensions were then diluted with FACS binding buffer and 10 µl of 50 µg/mL of Propidium Iodide (Sigma-Aldrich, Inc, St Louis, MO, USA) was added before acquiring samples. Cell debris was excluded by scatter gating (forward vs. side scatter). Approximately 50,000 events were collected per sample using CyAn ADP FACS cytometer (Beckman Coulter, Inc., CA, USA) and data was analysed with FloJo software (Tree Star, Inc, Oregon, USA). The percentage of apoptotic cells is expressed as the double positive cell population (AnnexinV+PI) and pre-apoptotic cells stained only for AnnexinV.

### γGT enzymatic and competition assays

For determination of γGT activity, ∼10^9^ bacteria were suspended in 400 µL of reaction buffer containing 1 mM of L-γ-glutamyl-p-nitroanilide (gGpNA) as described previously [6]. Additionally, culture supernatants, containing 50 µg/mL protein were analysed by diluting 50 µL of supernatant in 150 µL of reaction buffer. The kinetic parameters for the enzymatic reaction of the recombinant proteins were measured by following the cleavage of gGpNA. To determine the exchange rate of the substrate to 4-p-nitroaniline, the reaction was monitored at 405 nm and calculated by using the reported extinction coefficient of 8800 M/cm. Enzymatic activity was measured in 0.1 M Tris HCl with a pH range from 5.0 to 9.0, 20 mM glycyl-glycine and gGpNA at a concentration range from 1 µM to 2.5 mM. The experiments were carried out at 37°C for 30 min using a Mithras LB940 Well-Reader. The initial rates were determined at each gGpNA concentration and entered in the Michaelis-Menten equation. To investigate the affinity of the postulated γGT-substrates (i.e. L-Glutamine, D-Glutamine, L-Glutamic acid and Glutathione), we made a competition experiment in which the hydrolysis and transpeptidation of gGpNA was analyzed by measuring the absorption at 405 nm in the presence of γGT-substrates. For this assay we used 250 ng of the Hb-γGT or Hp-γGT per reaction with 100 µM gGpNA, 10 mM GlyGly and the substrates in a concentration-range between 0–5 mM in 100 mM Tris/HCl pH 8.0. The measurement was performed for 15 min at 37°C in a total volume of 200 µl.

## Supporting Information

Figure S1 **CD spectrum and photomultiplier voltage for recombinant Bgh1.** (A) CD spectrum for recombinant Bgh1 (black line), the predicted spectrum for Bgh1 obtained by K2D3 (red line) and lysozyme (blue line). The spectrum for lysozyme was taken from the Protein Circular Dichroism Data Bank (http://pcddb.cryst.bbk.ac.uk/). (B) Photomultiplier voltage of the spectrum for recombinant Bgh1.
(TIF)

Figure S2 **Rate of gGpNA turnover of culture supernatant (50 µg/mL total protein) of** ***Helicobacter bilis*** **wild-type CCUG 23435 (WT) and** ***H. bilis*** **Δ** ***ggt*** **MR9 (Δ** ***ggt*** **).** The white bars show the results for untreated culture supernatants, while the black bars show the results after treatment of culture supernatants with 50 µM of Acivicin.
(TIF)

Figure S3 **γGT-activity competition with glutathione, L-/D-Glutamine and L-Glutamic Acid.** Error bars in the graphs were calculated as SEM. The analysis was performed using Prism4 v4.03 (GraphPad Software). Inhibitory effect with various amounts of substrate analogues on (A) *Helicobacter bilis* γGT (Hb-γGT) and (B) *Helicobacter pylori* γGT (Hp-γGT).
(TIF)

Figure S4 **Apoptosis analysis of AGS cells after 24 hour treatment with γGT recombinant proteins.** AnnexinV and PI staining of AGS cells analysed by Flow cytometer treated with 0.5, 0.25 and 5 µg/mL of recombinant proteins from *Helicobacter bilis* (Hb-γGT), and *Helicobacter pylori* γGT (Hp-γGT), compared to untreated cells and cells treated with staurosporin.
(TIF)
