# Supplementary figures and images for: Evidence for Conserved Function of γ–Glutamyltranspeptidase in Helicobacter Genus
Source: PLoS One. 2012 Feb 14;7(2):e30543. doi: 10.1371/journal.pone.0030543 (PMC3279353; doi:10.1371/journal.pone.0030543)

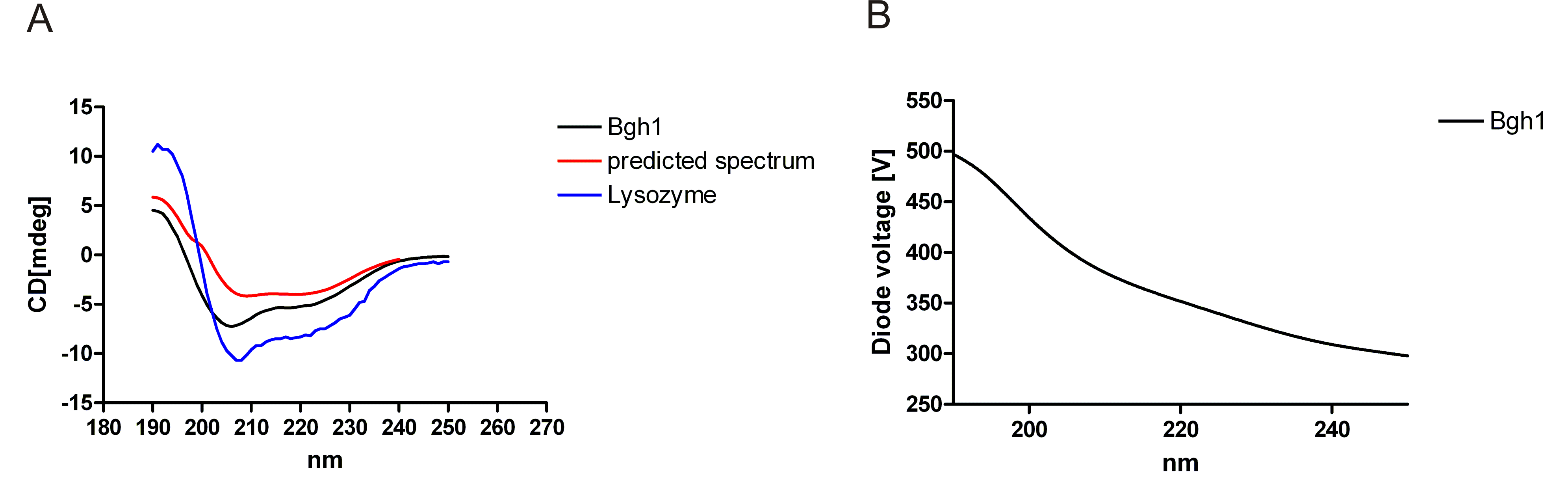

Supplement: Figure S1 — CD spectrum and photomultiplier voltage for recombinant Bgh1. (A) CD spectrum for recombinant Bgh1 (black line), the predicted spectrum for Bgh1 obtained by K2D3 (red line) and lysozyme (blue line). The spectrum for lysozyme was taken from the Protein Circular Dichroism Data Bank (http://pcddb.cryst.bbk.ac.uk/). (B) Photomultiplier voltage of the spectrum for recombinant Bgh1. (TIF) [file pone.0030543.s001.tif]

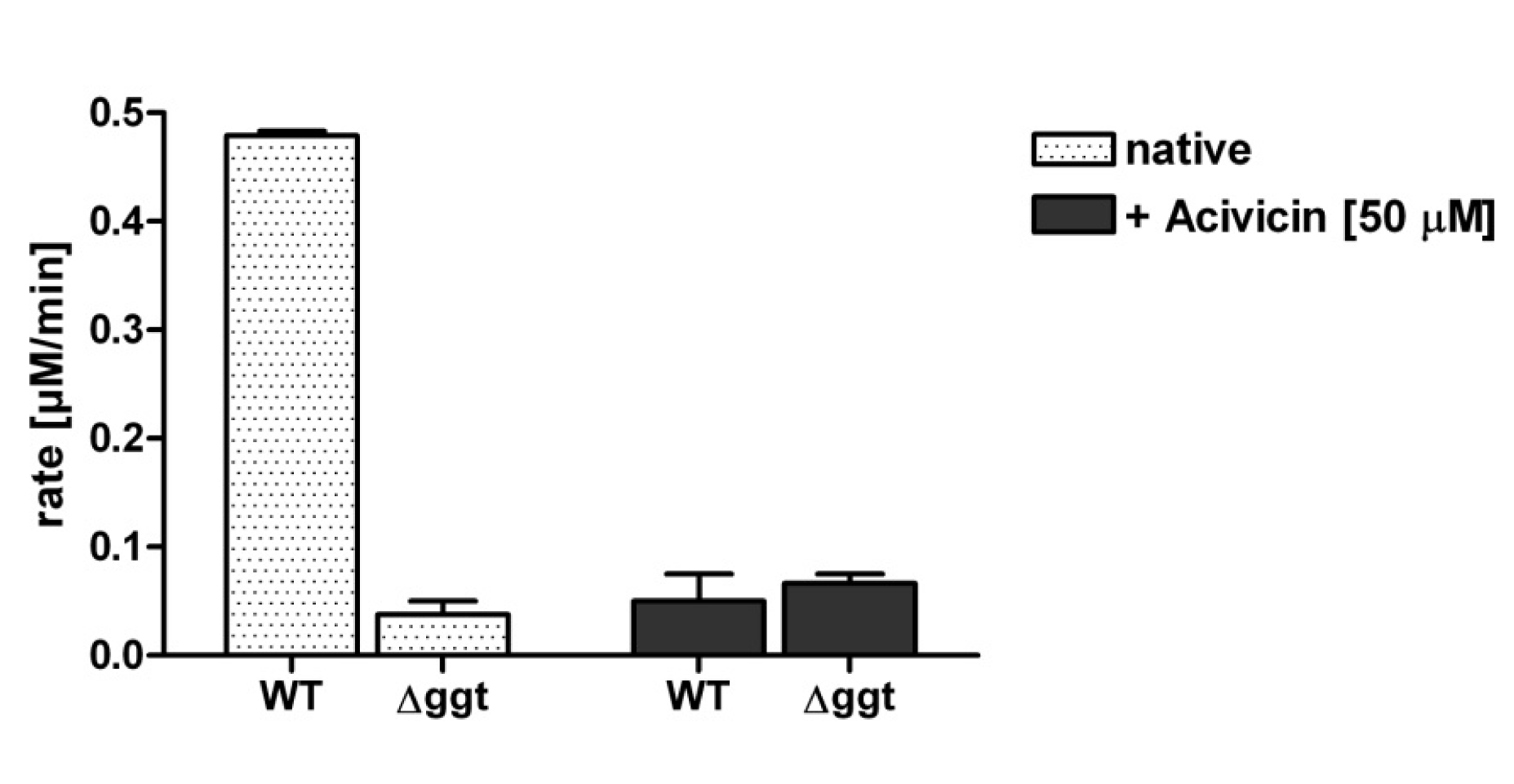

Supplement: Figure S2 — Rate of gGpNA turnover of culture supernatant (50 µg/mL total protein) of Helicobacter bilis wild-type CCUG 23435 (WT) and H. bilis Δ ggt MR9 (Δ ggt ). The white bars show the results for untreated culture supernatants, while the black bars show the results after treatment of culture supernatants with 50 µM of Acivicin. (TIF) [file pone.0030543.s002.tif]

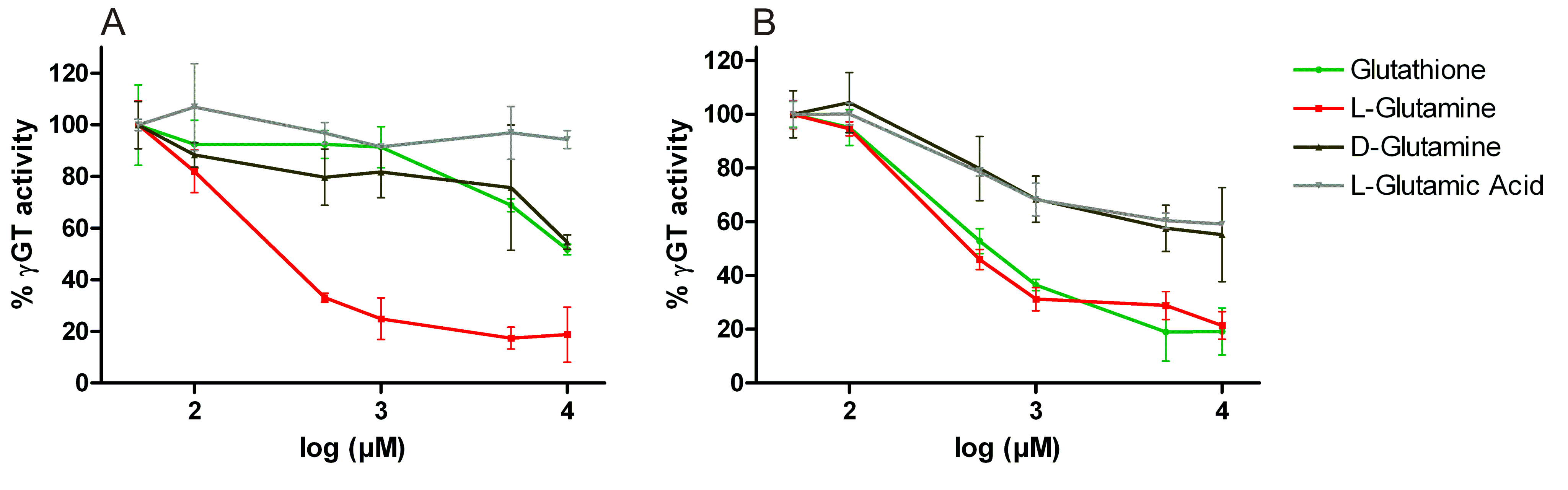

Supplement: Figure S3 — γGT-activity competition with glutathione, L-/D-Glutamine and L-Glutamic Acid. Error bars in the graphs were calculated as SEM. The analysis was performed using Prism4 v4.03 (GraphPad Software). Inhibitory effect with various amounts of substrate analogues on (A) Helicobacter bilis γGT (Hb-γGT) and (B) Helicobacter pylori γGT (Hp-γGT). (TIF) [file pone.0030543.s003.tif]

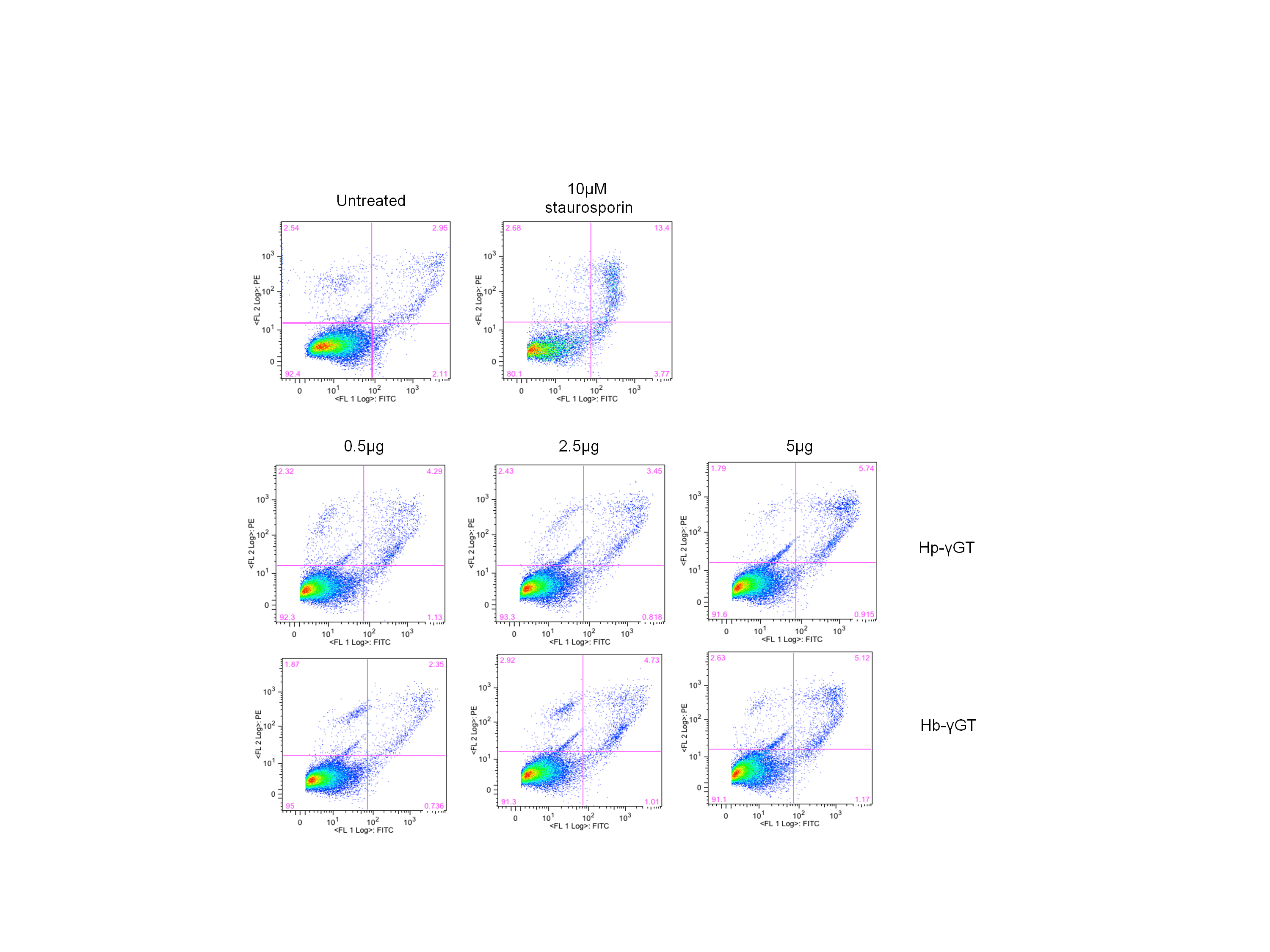

Supplement: Figure S4 — Apoptosis analysis of AGS cells after 24 hour treatment with γGT recombinant proteins. AnnexinV and PI staining of AGS cells analysed by Flow cytometer treated with 0.5, 0.25 and 5 µg/mL of recombinant proteins from Helicobacter bilis (Hb-γGT), and Helicobacter pylori γGT (Hp-γGT), compared to untreated cells and cells treated with staurosporin. (TIF) [file pone.0030543.s004.tif]
